# The development of Chinese population genetics by Ruofu Du

**DOI:** 10.1007/s13238-016-0265-6

**Published:** 2016-04-19

**Authors:** Chongfeng Xu, Ziyuan Duan

**Affiliations:** Institute of Genetics and Developmental Biology, Chinese Academy of Sciences, Beijing, 100101 China

As a scientist, Professor Ruofu Du enjoyed an outstanding reputation both at home and abroad. For more than half a century, he devoted himself to the advancement of Chinese national anthropology and human population genetics, always keeping pace with global development trends of human genetics. As one of the founders of population genetics in China, Prof. Du promoted the human genome diversity project of China, and established the Chinese National Immortalized Cell Bank in Beijing, which is the most diverse population cell bank in China (http://lac.genetics.ac.cn/zhysxbk/ShowClass.asp?ClassID=17). Prof. Du compiled several books during his research career which have been widely used in the study of the basic theories of Chinese population genetics research. In 2014 Prof. Du won the “Anthropology of Lifetime Achievement Award”, which is sponsored by the Anthropological Association of Shanghai, to recognize those scientists in the field of anthropology who have made a significant and outstanding contribution the anthropology, for his 60 years of research in this field.

After receiving his PhD degree from the Soviet Union Lenin Glennon College, Du returned to China in 1958, when he was 28 years old. There undertook a project to assess the effect of ionizing radiation on mammals and human genetics. Based on the research results of a survey into a population who were exposed to radiation in China, he provided direct evidence about the influence on human genetics of ionizing radiation (Ruofu Du, 1964). Subsequently, the Health Department modified the permissible irradiation dose of exposure to professional radiation staff of our country according to Du’s proposal.

Prof. Du realized the scarcity of research data on human genetics in our country during this survey, and resolved to try his best to contribute in this field. Remarkably, Prof. Du continued his research work during the 10 years Cultural Revolution even though most of scientific research was forced to stop at that time. Meanwhile, he translated and published three important guiding books, *The Radiation Genetics and Breeding of Crop*, *Handbook of Mutation Breeding* and *Molecular Genetics* during this period of unrest, with the cooperation of Mr. Jike Yang (杨纪珂).

In 1978 China began to introduce social reform and opening-up policies, after leading an academic visit to Australia, Prof. Du realized that Chinese research work had lagged far behind their international counterparts. When someone asked him, “What are you going to do when you go back home?” He said: “I think human genetics is the most backward in the field of genetics in our country. There has been a great deal of research into animals and plants, however as for research into human genetics, there are 56 ethnic groups and many special populations in China, but there is few related research about that, it is pity that we leave such a wealth of population genetic resources alone”. Thus, Du first proposed to carry out the study of human population genetics in China. Just as he said, “research of human population genetics is the need of the society and sciences, and that is my persistent goal.”

In 1979 Prof. Du and his human population genetics research group in Institute of Genetics, Chinese Academy of Sciences, which was established in Beijing, visited many remote regions all over the country and carried out a systematic investigation into the physical anthropology and cultural anthropology of the 56 ethnic groups in China, including extensive sampling and analysis on several indicators of human population genetics (Fig. 1). Summing up the investigation, Prof. Du compiled two books, *Chinese Nations* and *Chinese National Population Genetics* (Ruofu Du, 1994, 2004). *Chinese Nations* is not only widely used as a reference book in the National Committee at all levels of our country and autonomous region, but also for ethnology, anthropology and population genetics researchers. *Chinese National Population Genetics* has been widely used as an introduction into the theories of genetics. Prof. Du stressed that the aim of Chinese population genetics research is to study genetic structure, the similarities and differences, as well as changes among the population of all ethnic groups in China, so as to reveal the consanguineous relationship between populations to discover human migration and evolution routes, as well as susceptibility to various diseases and drugs and responses to different environmental factors. Prof. Du was the first to carry out such an extensive and detailed investigation into the various genetic indexes of the Chinese people (Ruofu Du, 1987, 1997).

**Fig. 1:**
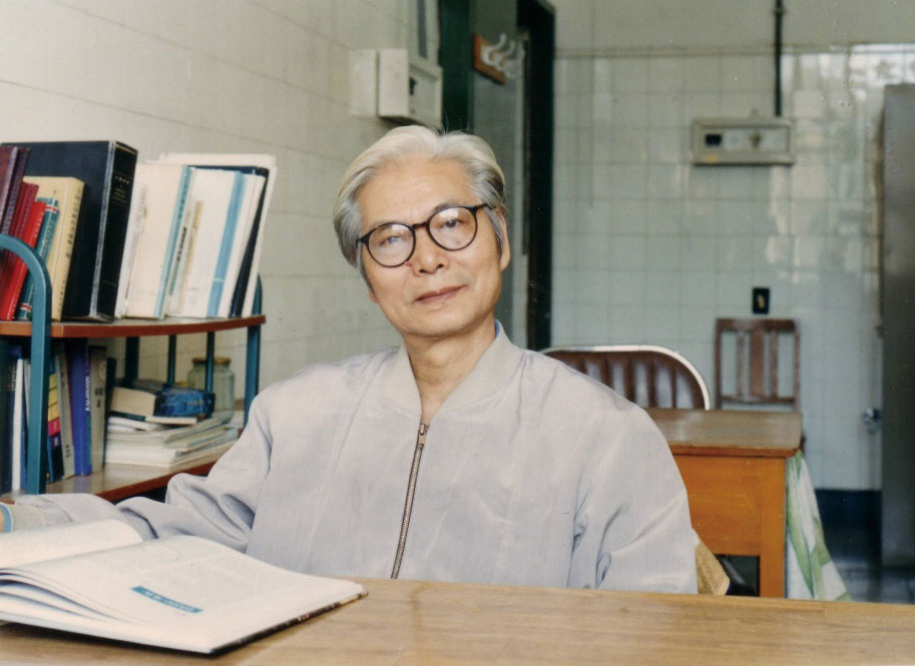
**Professor Ruofu Du at office**

Prof. Du was the first to propose to carry out the human genome diversity research program in China and to put forward the establishment of the Chinese national immortalized cell bank project. The International Human Genome Project (HGP) was launched in 1990, and Human Genome Diversity Plan (HGDP) Executive Committee was established in many countries in 1991. The Human Genome Diversity Research project of China which is led by Prof. Du is currently being conducted at a level equal to those of international peers. During this period in 1990s, Prof. Du served on the international human genome diversity Standing Committee and the “Chinese National Immortalized Cell Bank” which is led by Prof. Du, joined the international human genome diversity project. Prof. Du is one of the authors in *Science* article “human genome diversity cell line program (HGDP)” which is corresponded by Cavalli-Sforza L.L. (Cann et al., 2002). The cell lines provided by Chinese National Immortalized Cell Bank ensure the richness and integrity of the program (Fig. 2).

**Figure 2:**
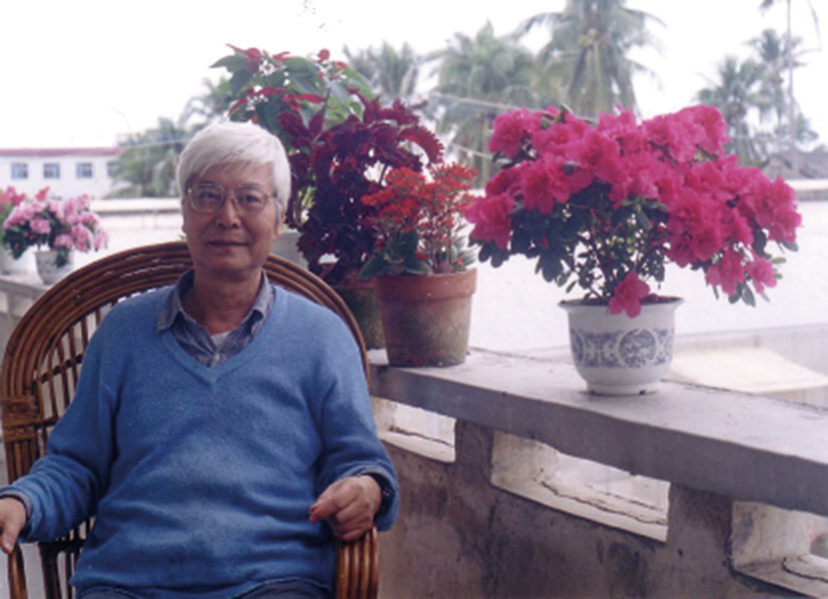
**Professor Ruofu Du in Beijing**

With over 20 years of research experience and the help of several minority scholars, Prof. Du recently took the time to compile a new book *Family Names of Chinese Minorities*, published by the Ethnic Publishing House in 2011. Famous professor Quangen Wang of Beijing Normal University gave a high evaluation to this book (Quangen Wang, 2010), which is the first monograph in this field.

Prof. Du is keen on social work and academic activities in human genetics. He served as deputy editor in chief of *Hereditas* and *Journal of Genetics and Genomics*, also on the editorial board of *Journal of Anthropology*, *Chinese Journal of Medical Genetics* and *Foreign Medical Sciences* (*Section of Genetics*), *Human Biology* and so on.

Prof. Du has made outstanding contributions in many fields of anthropology. Just as he expected (Ruofu Du, 1987, 1997), the immortalized cell bank of ethnic groups in China is gradually expanding, and plays an increasingly large role in supporting the basic research in many fields, including genetics, evolution, disease pathology, drug susceptibility, etc.

## References

[CR1] Cann HM, De Toma C, Cazes L, Legrand MF, Morel V, Piouffre L, Bodmer J, Bodmer WF, Bonne-Tamir B, Cambon-Thomsen A, Chen Z (2002). A human genome diversity cell line pane. Science.

[CR2] Du R (1964) The genetic effects of ionizing radiation on mammals and humans. Atomic Energy Sci Technol 03:286–305. (杜若甫. (1964). 电离辐射对哺乳动物和人类的遗传学效应《原子能科学技术》1964 年第3期.286–305页.)

[CR3] Du R (1987) Research on human population genetics in China. Bull Biol 32(7):9–11.(杜若甫(1987).我国的人类群体遗传学研究.生物学通报.1987 年第32卷第7期9–11页).

[CR4] Du R (1994). Chinese nations (Chinese and English Edition).

[CR5] Du R (1997) Opinions about the research of human genome diversity in China. Bull Chin Acad Sci 6:398–402.(杜若甫.(1997). 对开展中国人类基因组多样性研究的思考《中国科学院院刊》1997 年第6期.398–402页.)

[CR6] Du R (2004). Chinese national population genetics.

[CR7] Wang Q (2010) The development and deepening of the research on the surname of the minority nationalities in China. China Book Rev 1:116–119. (王泉根. (2010). 中国少数民族姓氏研究的开拓与深化《中国图书评论》2010.1:116–119.)

